# Analysis of disease-associated objects at the Rat Genome Database

**DOI:** 10.1093/database/bat046

**Published:** 2013-06-21

**Authors:** Shur-Jen Wang, Stanley J. F. Laulederkind, G. T. Hayman, Jennifer R. Smith, Victoria Petri, Timothy F. Lowry, Rajni Nigam, Melinda R. Dwinell, Elizabeth A. Worthey, Diane H. Munzenmaier, Mary Shimoyama, Howard J. Jacob

**Affiliations:** ^1^Rat Genome Database, Human and Molecular Genetics Center, ^2^Department of Physiology, ^3^Department of Pediatrics and ^4^Department of Surgery, Medical College of Wisconsin, 8701 Watertown Plank Road, Milwaukee, WI 53226, USA

## Abstract

The Rat Genome Database (RGD) is the premier resource for genetic, genomic and phenotype data for the laboratory rat, *Rattus norvegicus*. In addition to organizing biological data from rats, the RGD team focuses on manual curation of gene–disease associations for rat, human and mouse. In this work, we have analyzed disease-associated strains, quantitative trait loci (QTL) and genes from rats. These disease objects form the basis for seven disease portals. Among disease portals, the cardiovascular disease and obesity/metabolic syndrome portals have the highest number of rat strains and QTL. These two portals share 398 rat QTL, and these shared QTL are highly concentrated on rat chromosomes 1 and 2. For disease-associated genes, we performed gene ontology (GO) enrichment analysis across portals using RatMine enrichment widgets. Fifteen GO terms, five from each GO aspect, were selected to profile enrichment patterns of each portal. Of the selected biological process (BP) terms, ‘regulation of programmed cell death’ was the top enriched term across all disease portals except in the obesity/metabolic syndrome portal where ‘lipid metabolic process’ was the most enriched term. ‘Cytosol’ and ‘nucleus’ were common cellular component (CC) annotations for disease genes, but only the cancer portal genes were highly enriched with ‘nucleus’ annotations. Similar enrichment patterns were observed in a parallel analysis using the DAVID functional annotation tool. The relationship between the preselected 15 GO terms and disease terms was examined reciprocally by retrieving rat genes annotated with these preselected terms. The individual GO term–annotated gene list showed enrichment in physiologically related diseases. For example, the ‘regulation of blood pressure’ genes were enriched with cardiovascular disease annotations, and the ‘lipid metabolic process’ genes with obesity annotations. Furthermore, we were able to enhance enrichment of neurological diseases by combining ‘G-protein coupled receptor binding’ annotated genes with ‘protein kinase binding’ annotated genes.

**Database URL:** http://rgd.mcw.edu

## Introduction

The Rat Genome Database (RGD; http://rgd.mcw.edu) provides a comprehensive catalogue of genes, quantitative trait loci (QTL) and strains, with associated biological data for the laboratory rat, *Rattus norvegicus* (1). The curated data in RGD are presented in an organized structure through the use of controlled vocabularies or ontologies. An ontology is a controlled, standardized vocabulary of well-defined terms with specified relationships between them. Ontologies enable accurate and consistent data sharing between data sources, thus encouraging the use and exchange of publicly available data. The first implemented ontology at RGD was the Gene Ontology (GO). GO uses controlled vocabularies to describe gene products in three aspects: biological process (BP), cellular component (CC) and molecular function (MF) (2). Over time the ontologies used at RGD have grown in number to provide more comprehensive annotation of data objects (3, 4). Currently, the RGD disease ontology (RDO) [derived from the ‘merged disease vocabulary’ (MEDIC) (5)], the GO, the mammalian phenotype ontology (6) and RGD’s pathway ontology (7) are used to annotate genes at RGD.

Besides rat data, RGD also contains manually curated human QTL and manually curated human and mouse disease and pathway annotations. This is to facilitate human disease research where combined use of model organisms and clinical studies is essential. Much of the disease data are organized in ‘portal’ format in which curated objects related to specific disease areas are integrated. Currently, RGD has established seven disease portals—the cancer portal, the cardiovascular disease portal, the diabetes portal, the immune and inflammatory disease portal (referred as the immune disease portal hereafter), the neurological disease portal, the obesity/metabolic syndrome portal and the respiratory disease portal.

In addition to describing biological data with ontologies/controlled vocabularies, RGD has also developed and adapted a variety of online tools to facilitate curation (8) and assist researchers in analyzing their data (9). Among these tools, RatMine (http://ratmine.mcw.edu/ratmine/begin.do), built on ‘InterMine’ technology (10), provides flexible options for searching, extracting and using data from RGD and other sources such as Ensembl, UniProKB and KEGG. RatMine allows researchers to view multiple ontology enrichments simultaneously when analyzing lists of objects. Using RatMine as a principle analysis tool, we analyzed disease-associated rat strains, QTL and genes across the RGD disease portals. To corroborate the RatMine results, we analyzed the same data with the DAVID functional annotation tool (11).

## Methods

### Disease curation

The RDO (http://rgd.mcw.edu/rgdweb/ontology/view.html?acc_id=RDO:0000001#s) derived from MEDIC (5), is used to annotate data objects with disease terms. MEDIC, developed at the Comparative Toxicogenomics Database, is a structured disease vocabulary with a combination of OMIM (Online Mendelian Inheritance in Man, http://www.omim.org/) and MeSH (Medical Subject Headings, http://www.nlm.nih.gov/mesh/2012/mesh_browser/MBrowser.html) disease terms. The RDO is an extension of MEDIC achieved by adding new terms and additional parent relationships to existing terms. A current obo format RDO file can be accessed and downloaded from the RGD ftp site (ftp://rgd.mcw.edu/pub/ontology/disease/).

### QTL and strains

Rat strain and QTL information are collected from research publications, as well as direct submissions from individual researchers and rat breeders worldwide. RGD has specific searches saved at NCBI (http://www.ncbi.nlm.nih.gov/pubmed) that retrieve newly published research articles every week. Mostly these searches are based on different types of strains, different ways of characterizing QTLs in the literature, different QTL names and also on the names of specialized rat researchers. During the process of curation, nomenclatures of strains and QTL are determined at RGD according to the guidelines laid out by the International Committee on Standardized Genetic Nomenclature for Mouse and Rat Genome and Nomenclature Committee (http://rgd.mcw.edu/nomen/nomen.shtml). RGD curators manually annotate both human and rat QTL with the RDO and the mammalian phenotype ontology (6).

### Genes

Disease annotations to genes are manually curated from publications retrieved by targeted searches of genes and disease terms in PubMed. To have comprehensive coverage of genes in specific disease areas and also to prioritize disease curation, a ranked gene list is generated for each portal by searching multiple human disease databases (for example, PhenoPedia—http://hugenavigator.net/HuGENavigator/startPagePhenoPedia.do, GeneCards—http://www.genecards.org/index.shtml and Genetic Association Database—http://geneticassociationdb.nih.gov/) and weighing these disease-associated genes according to frequency of citation and source of information (for example, manually curated data from PhenoPedia or Genetic Association Database are weighted more than automated annotations from GeneCards). The disease terms used to search the various human disease–gene databases to establish the targeted gene lists are the same terms curators use to search PubMed for comprehensive gene–disease information.

### Genome browsers

In the genomic analysis, two RGD customized genome browsers, the Rat Genome Browser (http://rgd.mcw.edu/fgb2/gbrowse/rgd_904/) and the Human Genome Browser (http://rgd.mcw.edu/fgb2/gbrowse/human_36_3/), were accessed from the ‘Genome Tools’ icon on the RGD home page (http://rgd.mcw.edu/). Both browsers are loaded with ‘Disease Related Tracks’ that can be selected from the ‘Select Tracks’ tab in the browser page. In this manuscript, the cardiovascular and obesity disease genes or QTL were defined as genes or QTL downloaded from two disease tracks, ‘cardiovascular diseases’ and ‘nutritional and metabolic diseases’.

### VCMap (http://animalgenome.org/VCmap/)

The synteny searches in this manuscript were performed in VCMap accessible from ‘Genome Tools’ on the RGD home page. Rat chromosome 2 was used as the Backbone to search for human syntenic regions and human chromosome 4 was used as the Backbone to search for rat syntenic regions. The rat genome assembly v3.4 and the human genome assembly GRCH37.p5 were used at the time of analysis.

### RatMine (http://ratmine.mcw.edu/ratmine/begin.do)

Disease-associated genes, QTL and strains were retrieved from disease portals and analyzed using RatMine. Because most of the analysis tools or widgets were designed for gene analysis, limited analysis for strains and QTL was performed. Two enrichment widgets (automated software modules), GO enrichment and disease ontology enrichment, were used to compare GO enrichment patterns among disease portal genes and disease enrichment patterns among rat genes annotated to selected GO terms. The Holm–Bonferroni method was chosen for multiple hypothesis test correction. The enrichment tables in Figure 3 were set to display terms with *P* > 0.05. The enrichment *P*-values were converted into ‘–Log *P*-value’ for comparison in Figure 4. If the *P*-value was > 0.05 (or ‘–Log *P*-value’ was <1.3) there was no significant enrichment of that ontology term for the disease genes in comparison with all rat genes in RGD. A greater value for ‘–Log *P*-value’ means more enrichment as compared with a smaller value. The *P*-value was calculated using the hypergeometric distribution. Four parameters were used to calculate each *P*-value:

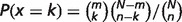



n = the number of objects in the gene list

N = the number of objects in the reference population (rat)

k = the number of objects annotated with this term in the gene list

m = the number of objects annotated with this term in the reference population (the whole rat genome is used as the reference population.)

### DAVID functional annotation tool

The DAVID 6.7 (The **D**atabase for **A**nnotation, **Vi**sualization and **I**ntegrated **D**iscovery) functional annotation tool (http://david.abcc.ncifcrf.gov/) (11) was used to analyze disease-associated genes retrieved from RGD disease portals. The DAVID functional annotation cluster tool groups genes based on their associated GO annotations. The related terms are clustered into groups with enrichment scores calculated from their EASEScore, the modified Fisher Exact *P*-value (12). Users can choose different ontology levels to group enriched terms. For example, ‘BP level 1’ uses the most general BP categories such as ‘biological process’ to group terms, whereas ‘BP level 5’ uses more specific BP categories such as ‘immune response’. There are six stringency levels (custom, lowest, low, medium, high and highest) available for displaying the results table. To see more specific clusters among terms, level 5 was used to analyze disease-associated genes from RGD, and medium stringency was selected for displaying results.

## Results

### Strain analysis

The seven current disease portals consolidate disease-related genes (rat, human, mouse), QTL (rat, human) and rat strains along with data associated with these objects. The analyses in this work focused on rat data from six representative portals: cancer, cardiovascular disease, immune disease, neurological disease, obesity/metabolic syndrome and respiratory disease (Table 1). The diabetes portal and the obesity/metabolic syndrome portal shared significant amounts of data. There was >90% overlap in genes, strains and QTL between the two portals. Thus, only the obesity/metabolic syndrome portal was chosen for analysis.

**Table 1.** Disease-associated rat genes, QTL and strains in the RGD disease portals^a^

RGD disease portal	Genes	QTL	strains
Cancer portal	651	74	55
Cardiovascular disease portal	924	491	310
Diabetes portal	1011	725	263
Immune and inflammatory disease portal	670	237	125
Neurological disease portal	1180	132	121
Obesity/metabolic syndrome portal	1049	729	267
Respiratory disease portal	328	1	4

^a^Accessed in May 2012.

**Figure 1.**
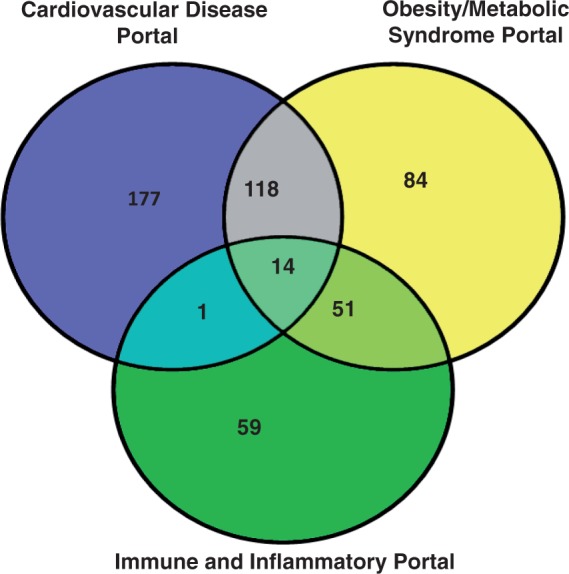
The rat strain distribution among three RGD disease portals. The numbers in each area represent the strain count of that section. Strain names are available from the RGD disease portals (http://rgd.mcw.edu/wg/portals).

The analysis of the three disease portals with the most rat strains is shown in Figure 1. The cardiovascular portal has 310 strains, and more than a third of them (132 strains) are also associated with the obesity/metabolic syndrome portal. This is not surprising because obesity is a major risk factor for the development of cardiovascular diseases (13, 14). More than half of the strains (65 out of 125) associated with the immune disease portal are also associated with the obesity/metabolic syndrome portal. The biological significance of this association may lie in the roles of adipokines in inflammation (15). These disease portal-associated rat strains are listed in the ‘Strains Info’ table in the individual portals (http://rgd.mcw.edu/wg/portals?100).

### QTL analysis

**Figure 2.**
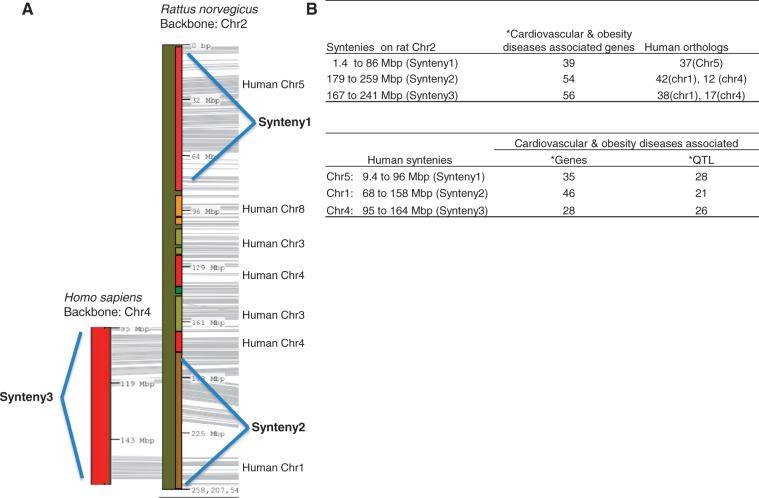
Cardiovascular and obesity diseases association of rat chromosome 2 and the human syntenies. Syntenic mapping of rat chromosome 2 to the human genome was performed using VCMap (http://animalgenome.org/VCmap/). (**A**) The two backbone chromosomes are labelled in Mbp. The chromosomal origins of syntenies are labelled to the right. (**B**) Rat and human syntenies are listed, with corresponding human orthologs for the rat syntenies, and disease-associated genes and QTL. (Asterisk) The cardiovascular and obesity diseases–associated genes (human and rat) and QTL (human) in the synteny were downloaded from ‘Disease Related Tracks’ (cardiovascular diseases and nutritional and metabolic diseases) from genome browsers at RGD. (rat: http://rgd.mcw.edu/fgb2/gbrowse/rgd_904/ and human: http://rgd.mcw.edu/fgb2/gbrowse/human_36_3/).

The highest counts of disease-associated QTL are in the cardiovascular disease, diabetes and obesity/metabolic syndrome portals (Table 1). All these disease-associated QTL are listed in the ‘QTLs Info’ table in the respective portal. There are 398 QTL associated with both the cardiovascular portal and the obesity/metabolic syndrome portal. These shared cardiovascular/obesity QTL are distributed across the genome with the greatest numbers on chromosomes 1, 2, 3 and 10 (Table 2). These four chromosomes also harbour the highest number of genes (Table 2). Chromosome 1, the largest rat chromosome, has the highest number of genes and QTL. Chromosome 2, although slightly smaller than chromosome 1, has only about half as many genes (52%) and QTL (60%) as chromosome 1. Despite having fewer genes and QTL, chromosome 2 has the highest number of cardiovascular/obesity QTL. The clustering of cardiovascular/obesity QTL on chromosome 2 suggests the importance of this chromosome in cardiovascular and obesity disease modelling in the rat. The cardiovascular and obesity disease association of human chromosomes homologous to this rat chromosome was examined. The two longest conserved human regions homologous to rat chromosome 2 correspond to the ends of the rat chromosome (Figure 2A). The rat synteny 1 (from 1.4 to 86 Mb) is homologous to human chromosome 5 (human synteny 1, from 9.4 to 96 Mb), and the rat synteny 2 (from 179 to 259 Mb) to human chromosome 1 (human synteny 2, from 68 to 158 Mb). These two rat syntenic blocks house 93 cardiovascular and obesity disease genes [data accessed January 2013, from the Rat Genome Browser (http://rgd.mcw.edu/fgb2/gbrowse/rgd_904/)]. The majority of human orthologs of these rat disease–associated genes are on human chromosomes 1 (42 genes), 4 (12 genes) and 5 (37 genes). Using human chromosome 4 as Backbone, we identified another region on human chromosome 4 (human synteny 3, from 95 to 164 Mb) homologous to rat chromosome 2 (rat synteny 3, from 167 to 241 Mb) (Figure 2A). All three human syntenic regions have genes and QTL associated with cardiovascular and obesity diseases (Figure 2B).

**Table 2.** Chromosome distribution of QTL associated with both the cardiovascular disease portal and the obesity/metabolic syndrome portal

Chromosome	Cardiovascular/ obesity QTL^a^	Total QTL	Total genes	Chromosome size(bp)
1	60	266	3975	267 910 886
2	61	159	2066	258 207 540
3	33	117	2272	171 063 335
4	15	142	1855	187 126 005
5	26	102	1799	173 096 209
6	10	75	1366	147 636 619
7	18	94	1884	143 002 779
8	19	90	1612	129 041 809
9	7	44	1025	113 440 463
10	36	165	2052	110 718 848
11	5	32	809	87 759 784
12	12	53	801	46 782 294
13	13	52	936	111 154 910
14	4	51	1029	112 194 335
15	8	53	1164	109 758 846
16	8	42	890	90 238 779
17	23	77	936	97 296 363
18	29	77	755	87 265 094
19	5	28	731	59 218 465
20	3	31	1017	55 268 282
X	3	22	1502	160 699 376

^a^Total 398 QTL were associated with both disesase portals.

The numbers of total QTL and genes on each chromosome were queried from the RGD QTL search (http://rgd.mcw.edu/rgdweb/search/qtls.html?100). The chromosome sizes were obtained from the rat genome browser (http://rgd.mcw.edu/fgb2/gbrowse/rgd_904/) using the rat genome v3.4 assembly.

### GO enrichment analysis of disease genes in RGD disease portals

Disease genes from rats were analyzed using the in-house data-mining tool RatMine (http://ratmine.mcw.edu/ratmine/begin.do). The DAVID functional annotation tool (http://david.abcc.ncifcrf.gov/home.jsp) was used to corroborate GO enrichment patterns obtained from RatMine. Disease-associated genes (from the cancer, cardiovascular disease, immune disease, neurological disease, obesity/metabolic syndrome and respiratory disease portals) were copied from the ‘Genes Info’ table in each portal (http://rgd.mcw.edu/wg/portals), and made into lists in the tool for GO enrichment analysis. The focus of gene analysis was on the cardiovascular disease and obesity/metabolic syndrome portals because these two portals were the most complete portals in terms of strains, QTL and genes.

**Figure 3.**
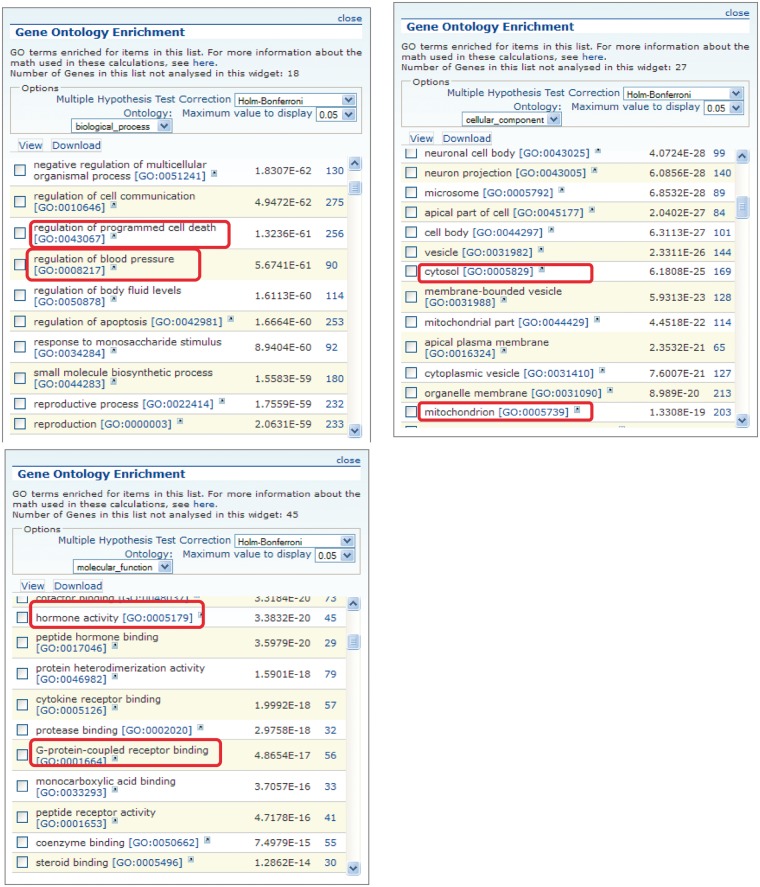
GO enrichment tables for the three GO aspects of genes associated with the obesity/metabolic syndrome portal. A total of 1049 rat genes associated with this disease portal were subjected to GO enrichment analysis in RatMine. Only the top portions of the enrichment tables are shown. Two GO terms selected from each GO aspect for comparison are highlighted.

**Figure 4.**
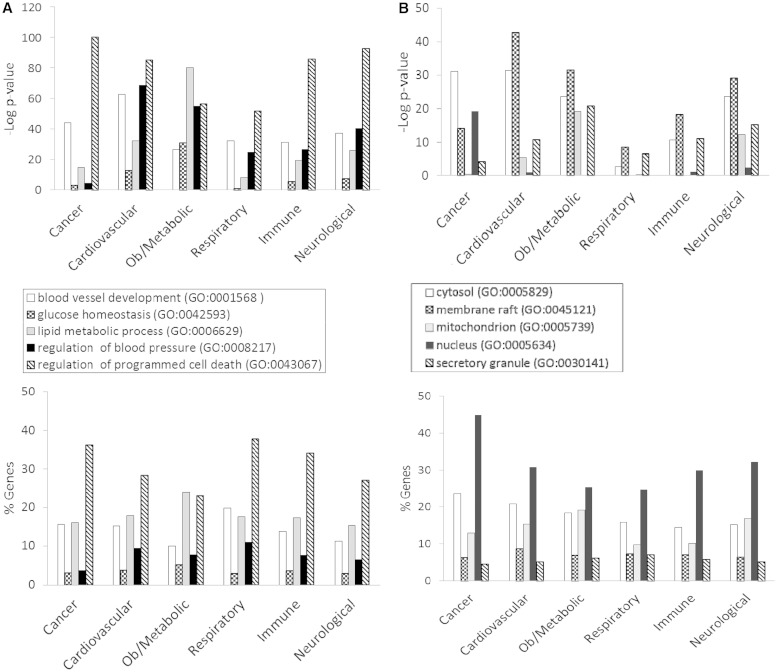

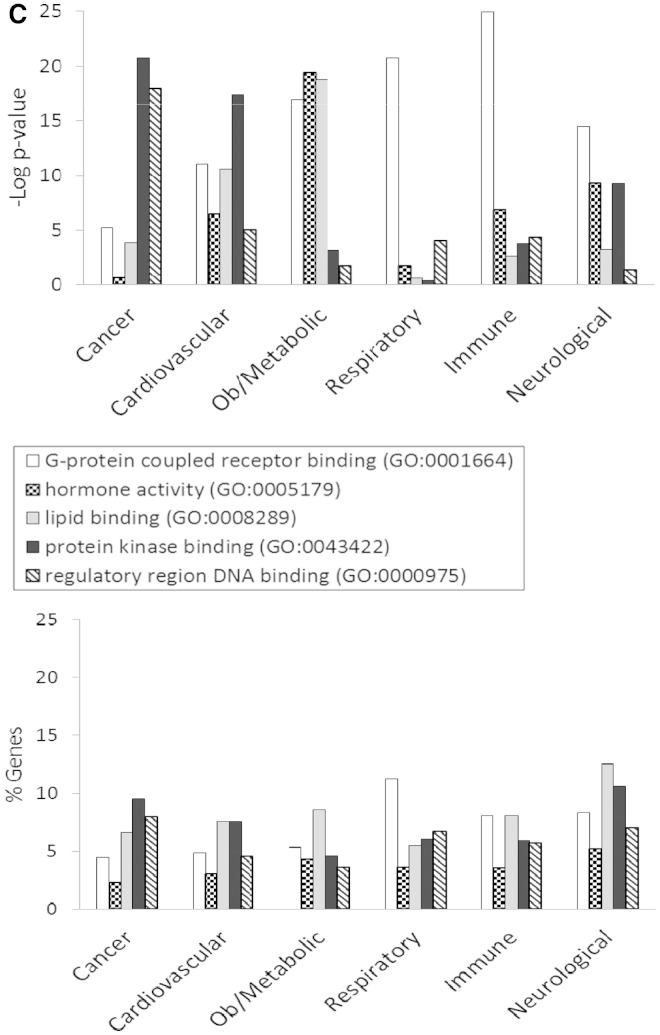
(**A**) The BP annotations of the disease-associated genes at RGD were subjected to enrichment analysis using RatMine. The enrichment *P*-values, presented as ‘–Log *P*-value’ are shown in the top panel, and the percentages of genes annotated with the relevant GO term and its children are shown in the bottom panel. The six RGD disease portals—cancer portal (cancer), cardiovascular disease portal (cardiovascular), obesity/metabolic syndrome portal (ob/metabolic), respiratory disease portal (respiratory), immune and inflammatory disease portal (immune) and neurological disease portal (neurological)—are listed across the x axis. (**B**) The CC annotations of the disease-associated genes at RGD were subjected to enrichment analysis using RatMine. The enrichment *P*-values, presented as ‘–Log *P*-value’ are shown in the top panel, and the percentages of genes annotated with the relevant GO term and its children are shown in the bottom panel. The six RGD disease portals—cancer portal (cancer), cardiovascular disease portal (cardiovascular), obesity/metabolic syndrome portal (ob/metabolic), respiratory disease portal (respiratory), immune and inflammatory disease portal (immune) and neurological disease portal (neurological)—are listed across the x axis. (**C**) The MF annotations of the disease-associated genes at RGD were subjected to enrichment analysis using RatMine. The enrichment *P*-values, presented as ‘–Log *P*-value’ are shown in the top panel, and the percentages of genes annotated with the relevant GO term and its children are shown in the bottom panel. The six RGD disease portals—cancer portal (cancer), cardiovascular disease portal (cardiovascular), obesity/metabolic syndrome portal (ob/metabolic), respiratory disease portal (respiratory), immune and inflammatory disease portal (immune) and neurological disease portal (neurological)—are listed across the x axis.

Figure 3 shows the GO enrichment tables of genes associated with the obesity/metabolic syndrome portal. The RatMine widgets listed 1603 BP terms, 113 CC terms and 140 MF terms. To make an informative comparison across disease portals, we first screened out high-level general terms such as ‘response to organic substance (GO:0010033)’, ‘negative regulation of multicellular organismal process (GO:0051241)’, ‘organ development (GO:0048513)’ and ‘localization (GO:0051179)’. These high-level terms give broad biological information that may not be informative to understand the roles of genes in diseases. Instead, GO terms that provided more specific information about gene products in each GO aspect were chosen for comparison across portals. Fifteen GO terms, five for each aspect, were selected to profile the six disease portals using their enrichment *P*-values and percentage of genes annotated with the selected GO terms. The enrichment *P*-values of each term were converted to ‘–Log *P*-value’ for comparing levels of enrichment among selected GO term/disease portal combinations (Figure 4). The percentages of genes annotated with a selected GO term are shown in the bottom panels to compare the prevalence of annotated genes in each disease portal. The distribution profile of ‘–Log *P*-value’ gives a better approximation of the importance of certain terms for a particular disease portal because it eliminates the relative chance occurrence of terms that can show up in the ‘% Genes’ of each portal annotated to each term.

Four BP terms (‘blood vessel development’, ‘glucose homeostasis’, ‘lipid metabolic process’ and ‘regulation of blood pressure’) were selected based on the high enrichment scores and their physiological relevance in cardiovascular and obesity diseases. The term ‘regulation of programmed cell death’ was selected for its high enrichment and frequent occurrence across all portals (Figure 4A). This common annotation serves as an internal reference for comparison. Among genes associated with obesity and metabolic syndromes, ‘lipid metabolic process’ was enriched more than ‘regulation of programmed cell death’. This demonstrated the unique importance of the lipid metabolism process in the Obesity/Metabolic Syndrome diseases. The BP term ‘blood vessel development’ was not just enriched in the cardiovascular disease portal, but also highly enriched across the other five portals (Figure 4A). This reflects the fact that blood vessel development is important in disease processes such as cancer (tumor angiogenesis) (16) and immune/inflammatory processes (17).

For CC terms, ‘nucleus’, ‘cytosol’ and ‘mitochondrion’ were selected to locate gene products in the nucleus, cytosol or organelle (mitochondrion), and ‘membrane raft’ and ‘secretory granule’ were selected to examine gene products localized in specific functional membrane domains and organelles. Among the selected CC terms, ‘cytosol’ was annotated to 15–23% of disease genes across portals. There were higher percentages (ranging from 24 to 45%) of disease genes annotated with ‘nucleus’ (Figure 4B). However, only cancer-associated genes were highly enriched with ‘nucleus’ annotations (–Log *P* = 19.2). Another CC annotation, ‘mitochondrion’, was most enriched in the obesity/metabolic syndrome and neurological portals, and was least enriched in the cancer, respiratory disease and immune portals. This indicates that the frequencies of mitochondrion annotations in these latter three disease portals were not any higher than across the whole set of rat genes at RGD.

Most MF terms shown in the enrichment tables are binding and activity terms (Figure 3); four binding terms, ‘regulatory region DNA binding’, ‘protein kinase binding’, ‘G-protein coupled receptor (GPCR) binding’ and ‘lipid binding’ were selected to analyze gene products binding to DNA, protein (kinase and receptor) and lipid. ‘Hormone activity’ was the only activity term selected for its high enrichment in the obesity/metabolic syndrome portal and it is not redundant with the other four selected binding terms. Among selected MF terms analyzed, ‘GPCR binding’ was the most enriched term in the immune, respiratory and neurological disease portals. ‘Protein kinase binding’ was most enriched in the cancer and cardiovascular disease portals. The obesity/metabolic syndrome portal was highly enriched with three MF terms: ‘GPCR binding’, ‘hormone activity’ and ‘lipid binding’ (Figure 4C).

### DAVID functional annotation tool analysis

In addition to the RatMine tool, we also used the DAVID functional annotation tool to examine GO enrichment of disease portal genes at RGD. The clustering feature of the DAVID tool combines genes into annotation groups based on the degree of their co-association, and ranks each group with a calculated enrichment score (11, 12). The group numbers are a reference for comparison within a gene list, and the scores can be compared across disease gene lists. The disease genes were grouped into clusters according to BP annotations and CC annotations; there was no enriched gene cluster among MF annotations. In the BP aspect, cancer portal genes were highly scored with ‘regulation of programmed cell death’ and ‘blood vessel development’, and these genes were clustered into ‘annotation cluster 1’ and ‘annotation cluster 3’, respectively (Table 3A). The obesity/metabolic syndrome portal genes annotated with these two BP terms were ranked in the 6th and 10th clusters with lower enrichment scores than those of the cancer portal (Table 3A).This pattern is similar to the GO enrichment shown in Figure 4A, where the obesity genes are less enriched in these two terms compared with cancer genes.

**Table 3.** GO enrichment analysis of genes in the RGD disease portals using the DAVID functional annotation tool (v.6.7)

A. Biological process					
RGD disease portal	Regulation of programmed cell death			Blood vessel development	
	Annotation cluster	Score		Annotation cluster	Score
Cancer	1	43.8	Cardiovascular	3	39.8
Cardiovascular	4	36.4	Cancer	3	34.1
Neurological	3	34.1	Neurological	10	22.2
Immune	1	32.5	Obesity/metabolic	10	20.1
Obesity/metabolic	6	23.0	Respiratory	2	19.5
Respiratory	4	17.9	Immune	11	14.4

B. Cellular component					
RGD disease portal	Nucleus			Mitochondrion	
	Annotation cluster	Score		Annotation cluster	Score
Cancer	1	15.6	Obesity/metabolic	3	14.3
Obesity/Metabolic	8^a^	3.26^a^	Neurological	4	7.2
Respiratory	5^a^	1.46^a^	Cardiovascular	8	2.8
Cardiovascular	12^a^	1.45^a^	Immune	10	1.3
Neurological	17^a^	0.80^a^	Cancer	10	1.0
Immune	13^a^	0.48^a^	Respiratory	9^a^	0.23^a^

The disease genes in each portal were grouped into clusters according to related annotations. Each group was ranked with a calculated enrichment score (score).

^a^Calculated from the enrichment of the child terms of the selected GO terms if the parent terms did not group into clusters.

Cancer portal genes annotated with ‘nucleus’ were grouped in ‘annotation cluster 1’ with the highest enrichment score of 15.6 (Table 3B) among the portals. Genes in the other five disease portals were less significantly annotated with ‘nucleus’ and were scored much lower. In RatMine analysis, obesity/metabolic syndrome portal genes were highly enriched with ‘mitochondrion’ annotations (–Log *P* = 19.2, Figure 4B). This enrichment was reproduced in clustering analysis where ‘mitochondrion’-annotated obesity/metabolic syndrome portal genes were in ‘annotation cluster 3’ with an enrichment score of 14.3 (Table 3B). As in the RatMine analysis, obesity/metabolic syndrome portal genes were followed by neurological disease portal genes and cardiovascular disease portal genes in DAVID analysis of ‘mitochondrion’ annotations.

### From GO to diseases

**Figure 5.**
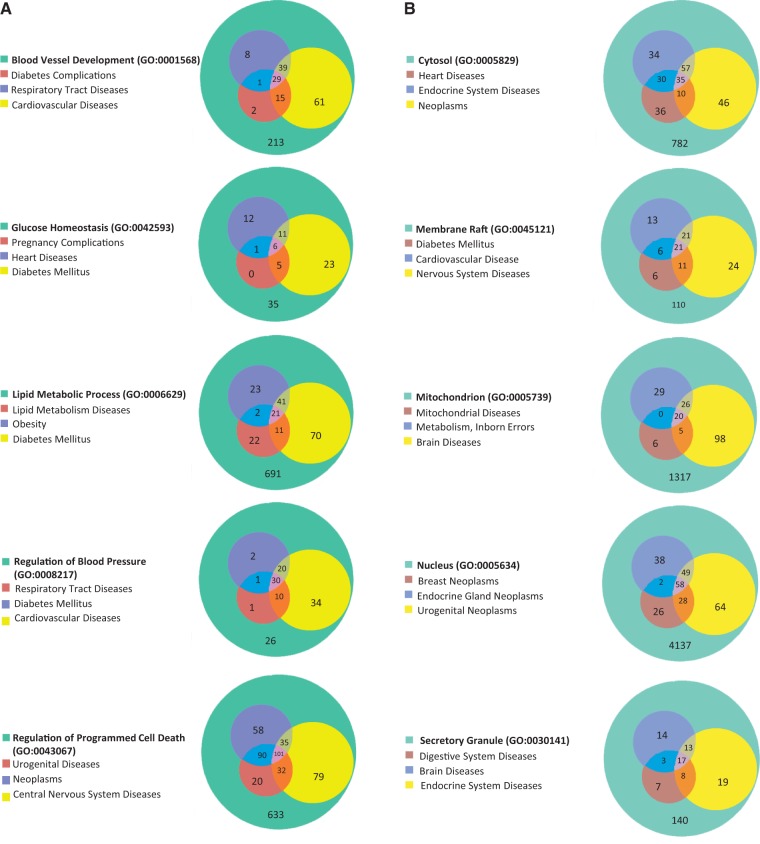

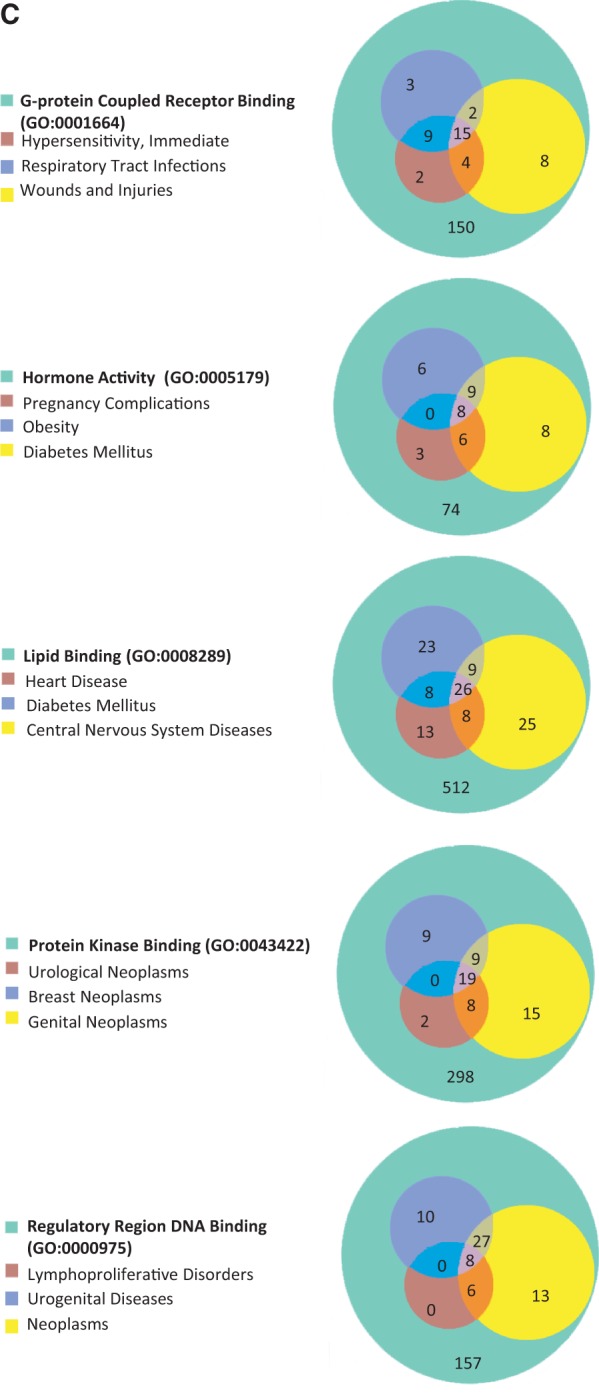
(**A**) The disease enrichment analysis of five BP-annotated gene lists. The three most enriched diseases from each gene list are presented in Venn diagrams. The numbers in each area represent the gene count of the section. (**B**) The disease enrichment analysis of five CC-annotated gene lists. The three most enriched diseases from each gene list are presented in Venn diagrams. The numbers in each area represent the gene count of that section. (**C**) The disease enrichment analysis of five MF annotated gene lists. The three most enriched diseases from each gene list are presented in Venn diagrams. The numbers in each area represent the gene count of that section.

The 15 GO terms used to analyze disease portals were then used as search parameters to retrieve all annotated rat genes from RatMine (http://ratmine.mcw.edu/ratmine/begin.do). These 15 GO term gene lists (see Supplementary Tables S1A–C for the gene lists generated from these GO terms) were then subjected to disease vocabulary enrichment using the ‘Disease Ontology Enrichment’ widget in RatMine. To have a better idea of specific disease term distribution among GO-annotated genes, disease terms were manually selected from enrichment tables, based on high-level representation in the disease vocabulary tree so as to represent most of the disease terms in the list. The three most enriched disease terms were selected for each GO term–annotated gene list, and the distribution of diseases among gene lists are displayed in Venn diagrams, with the total GO term–annotated genes represented by the largest circle in each diagram (Figure 5). Each of the other circles represent genes annotated to both the indicated disease term and GO term.

BP-annotated genes in general showed strong enrichment in diseases logically related to the GO terms. For example, both the ‘blood vessel development’– and ‘regulation of blood pressure’–annotated gene lists were enriched with ‘cardiovascular disease’ annotations, as well as the ‘glucose homeostasis’–annotated gene list with ‘diabetes mellitus’ annotations (Figure 5A). The ‘regulation of blood pressure’–annotated genes were also enriched in ‘diabetes mellitus’ annotations and ‘respiratory tract diseases’ annotations, and >94% of the genes associated with these two diseases were also associated with cardiovascular diseases. The close association of diabetes and cardiovascular diseases with genes involved in ‘regulation of blood pressure’ is predictable based on the common cardiovascular complications of diabetes and the association of hypertension with cardiovascular disease (18). Among ‘nucleus’-annotated genes, the most enriched three diseases are types of neoplasms (Figure 5B), which is reasonable because most cancers are caused by damaging alterations in biological processes such as transcription and replication occurring in the nucleus. Other neoplasm-enriched gene lists are the ‘regulatory region DNA binding’ list and ‘protein kinase binding’ list (Figure 5C). In the ‘regulatory region DNA binding’ gene list, all of the genes associated with ‘lymphoproliferative disorders’ are associated with ‘neoplasms’, which is also to be expected because lymphoproliferative disorders are similar to neoplasms by way of excessive cell proliferation and one would expect the same genes to be involved in both types of diseases. The prevalence of ‘neoplasms’ annotations in the ‘protein kinase binding’ list could be explained by the importance of growth factor pathways in cancers, with protein kinases playing key roles in regulating growth factor pathways.

### Enrichment of neurological diseases by combining gene lists

**Figure 6.**
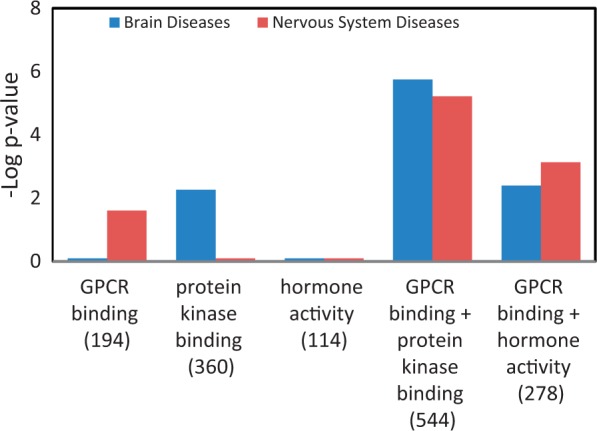
Enhanced enrichment of neurological diseases by combining MF term–annotated gene lists. The ‘GPCR binding’ gene list was combined with the ‘protein kinase binding’ gene list or the ‘hormone activity’ gene list. The enrichment *P*-values for these two diseases, shown as ‘–Log *P*-value’ were compared before and after combination. The gene count of each list is shown in parenthesis.

Table 4 is a summary of the Venn diagrams shown in Figure 5C. The three major diseases, enriched in each MF gene list, were charted accordingly into disease portals. For example, the ‘+++’ under the ‘protein kinase binding’ gene list indicates that all three enriched diseases belong to the cancer portal. We did not catalogue ‘wounds and injuries’ and ‘pregnancy complication’ because these two diseases were under parent terms belonging to multiple portals. The disease enrichment patterns of MF gene lists correlated well with the MF enrichment patterns (Figure 4C) of all disease portals except the neurological disease portal. The most enriched MF term in the neurological portal was ‘G-protein coupled receptor binding (GPCR binding)’, yet the high-level neurological disease term ‘nervous system diseases’ is not one of the three major diseases enriched in the list (Figure 5C, Table 4). Among diseases enriched in the ‘GPCR binding’ gene list ‘nervous system diseases’ is towards the bottom of the enriched list with a –Log *P* = 1.6 (*P* = 0.0246 in Supplementary Table S2). This suggests that while ‘GPCR binding’ is important in neurological diseases, many other diseases are more significantly associated with genes involved in GPCR binding. From the enrichment profile of the neurological disease portal (Figure 4C), we proposed to create candidate gene lists with enhanced enrichment in neurological diseases by combining the ‘GPCR binding’ gene list with the ‘hormone activity’ gene list or the ‘protein kinase binding’ gene list. The ‘hormone activity’ and ‘protein kinase binding’ annotations are the second and third most enriched among the five MF annotations compared in the neurological disease portal (Figure 4C), yet neurological diseases are not one of the three major diseases enriched on either list (Figure 5C and Table 4). The two combined gene lists, ‘GPCR binding + protein kinase binding’ and ‘GPCR binding + hormone activity’, are unions of genes annotated with either one term or both terms. Two disease terms, ‘brain diseases’ and ‘nervous system diseases’ were selected for comparing the enrichment of neurological diseases before and after combining gene lists. Before combining with other gene lists, only the term ‘nervous system diseases’ was enriched with a –Log *P* = 1.6 in the ‘GPCR binding’ gene list. The –Log *P* were improved to 5.2 and 3.1 after combining with the ‘protein kinase binding’ gene list and ‘hormone activity’ gene list, respectively. The term ‘brain diseases’, which is not in the enrichment diseases of the ‘GPCR binding’ list, showed enrichment in both combined lists (Figure 6).

**Table 4.** Summary of enriched disease categories from MF GO term–annotated gene lists

RGD disease portal	GPCR binding	Hormone activity	Lipid binding	Protein kinase binding	Regulatory region DNA binding
Cancer				**+++**	**++**
Cardiovascular			**+**		
Obesity/metabolic Syndrome		**++**	**+**		
Respiratory	**+**				
Immune	**+**				**+**
Neurological			**+**		

The disease term enrichments in Figure 4C were charted according to disease portal. Each** + **represents one disease term.

## Discussion

The RGD disease portals were established by targeted annotation of strains, QTL and genes associated with specific disease areas. Additional disease areas have been added periodically, and disease annotations are added on a regular basis. From these disease portals, we identified rat strains associated with multiple portals. The cardiovascular disease portal and the obesity/metabolic syndrome portal shared 132 strains, which represent <40% of the strains in each portal. This reflects the fact that cardiovascular disease is a major complication of obesity and the metabolic syndrome (13, 14). The strains highlighted with more than one disease portal in Figure 1 provide models to study the interaction of inflammation, obesity, metabolic syndrome and cardiovascular diseases. Therefore, one would expect some of the genes and QTL to be responsible for both cardiovascular and obesity diseases. In the cardiovascular disease portal, 81% of the disease-associated QTL were also associated with the obesity/metabolic syndrome portal. These cardiovascular/obesity QTL distribute mainly across four chromosomes with the highest number on chromosome 2. The involvement of chromosome 2 in cardiovascular diseases has been shown in Brown Norway rats by association with myocardial ischemia protection (19), and in spontaneously hypertensive rats by association with hypertension, glucose tolerance and dyslipidemias (20). We also examined how this cardiovascular and obesity association in rats may be applied to human disease study. Three conserved regions have been identified between rat chromosome 2 and human chromosomes 1, 4 and 5. The importance of these human syntenies in cardiovascular and obesity diseases was confirmed by the presence of disease-associated genes and QTL (Figure 2B). The rat has been widely used as an animal model in the study of human obesity and cardiovascular diseases. In-depth genomic analysis of rat chromosome 2 and related human chromosomes would lead to new insights into the genetic basis of cardiovascular/obesity diseases. The combination of rat expression QTL and data mining has identified potential disease genes associated with hypertension in human (21).

We performed an extensive ontology analysis of disease-associated genes at RGD. The GO enrichment patterns of selected terms varied among portals. With a focus on terms important in cardiovascular and obesity diseases, we found that cancer-associated genes exhibited an enrichment profile distinctly different from that of the obesity/metabolic syndrome portal. In CC enrichment patterns, the cancer-associated genes are enriched with ‘nucleus’ annotations, but not with ‘mitochondrion’ annotations. On the other hand, the obesity/metabolic syndrome-associated genes are enriched with ‘mitochondrion’ but not with ‘nucleus’ annotations. In MF enrichment patterns, the cancer genes are highly enriched in ‘protein kinase binding’ (–Log *P* = 20.8), and ‘regulatory region DNA binding’ (–Log *P* = 18), yet these two terms are not enriched as highly among obesity/metabolic syndrome-associated genes (–Log *P* = 3.1 and 1.8, respectively). However, the other three MF terms, ‘GPCR binding’, ‘hormone activity’ and ‘lipid binding’, with –Log *P* < 16 among obesity/metabolic syndrome associated genes, are less enriched among cancer-associated genes (–Log *P* > 5.3) (Figure 4C). Lipids would logically be associated with diseases of obesity and the dyslipidemia feature of the metabolic syndrome. ‘Mitochondrion’ annotations would be expected to be associated with obesity/metabolic syndrome-associated genes because of energy-related issues that arise with metabolic imbalances involving either lipids or glucose. However, for cancer development, most of the growth alteration events are happening in the nucleus and involve altered signalling pathways regulated by protein kinases as discussed earlier. The observed difference in GO enrichment patterns between the cancer and obesity/metabolic syndrome portals has future application in linking GO annotations to diseases. This utility has been further underscored by using the enriched terms to perform gene searches and analyzing the disease term enrichment of these annotated genes. We found that some annotations, especially in BP, were strongly associated with certain diseases. The ‘blood vessel development’ genes and ‘regulation of blood pressure’ genes are enriched with ‘cardiovascular diseases’ annotations, and ‘glucose homeostasis’ genes are enriched with ‘diabetes mellitus’ annotations. One would anticipate that to be the case if the genes were sufficiently curated with both the GO and the disease vocabulary because gene products associated with blood pressure and glucose homeostasis would be expected to be associated with derangement of those processes, as happens in cardiovascular disease and diabetes mellitus. The predictable linkage between related BP terms and cardiovascular diseases has also been demonstrated by others (22).

The individual GO term–annotated gene list and disease annotation relationships shown in Venn diagrams (Figure 5) are consistent with most of the GO enrichment patterns of disease portal genes (Figure 4) except in the MF term gene lists. The discrepancy is the low enrichment of neurological diseases in the individual gene lists of ‘GPCR binding’, ‘protein kinase binding’ and ‘hormone activity’. (The enriched diseases of MF gene lists are in Supplementary Table S2). We were able to enhance the enrichment of neurological diseases by combining two gene lists from the three most enriched MF-annotated gene lists (Figure 6). This demonstrates that combining gene lists annotated with disease-related GO terms is a valid method to create specific disease candidate gene lists. The ‘GPCR binding + protein kinase binding’ gene list showed the most enhanced *P*-value in analysis and might be considered as a synthesized candidate gene list for neurological diseases. In this list, there are 116 genes with neurological disease annotations and 428 genes without. Out of these 428 genes, there could be new neurological disease genes if similar diseases are caused by similar mechanisms. To decrease the number of candidate genes, we compared this synthesized gene list with published gene sets associated with neurological diseases. Gene sets from brain regions of Alzheimer’s Disease (AD) patients showed enrichment in annotations associated with neurons and synapse function (23). We found that the ‘GPCR binding + protein kinase binding’ gene list also showed enrichment in neuronal structures such as synapse, dendrite and neuron spine. There were DAVID enrichment clusters that overlapped between the AD-associated genes and the combined gene list. Some of the overlapping enrichment clusters were ‘phosphate metabolic process’, ‘phosphorylation’ (annotation cluster 2), and ‘regulation of synaptic transmission’, ‘regulation of synaptic plasticity’ (annotation cluster 5). Based on the similarity in enrichment pattern, it is highly possible that there are genes present in both lists that are important in AD or neurological diseases. There were nine genes in common between the ‘GPCR binding + protein kinase binding’ list and AD-associated genes (compared with 172 unique genes in the top 30 up- and down-regulated genes in three brain regions). Out of these overlapping genes, FOXO4, GRM5, RIMS1 and NELL2, were found to be associated with neurological diseases in recent publications [GRM5 (24–26), RIMS1 (27), FOXO4 (28), NELL2 (29)] though they were not yet annotated with any neurological disease terms in the database (RGD, rgd.mcw.edu, last accessed October 2012). So, by combining specific GO term–annotated gene lists and cross-checking with publications, we were able to decrease the number of candidate genes and predict candidate genes (FOXO4, GRM5, RIMS1 and NELL2) for neurological diseases. Similarly, to construct candidate gene sets major mental depression, Kao *et al.* have combined and processed genetic data to generate candidate genes for the disease (30), and the gene sets have been extensively analyzed with pathway and GO annotations (31).

Ontology enrichment has been used, by us and others, to functionally identify genes differentially expressed during physiological or pathological conditions. However, for complex diseases, such as cardiovascular diseases and neurological disorders, a conclusively defined set of disease-associated genes might not be yet available. Mining existing data and constructing candidate gene lists can be a reasonable approach to obtain gene sets for complex diseases. To provide easy access to gene-disease data for the research community, RGD has put major effort into disease curation by first generating prioritized disease-associated gene lists from major disease databases. The disease association of these gene lists is then verified by PubMed publications, and valid information is manually curated. Users can obtain disease-associated data from the RGD website, http://rgd.mcw.edu/wg/portals, and analyze data using the RGD RatMine tool or other web applications such as the DAVID functional annotation tool.

## Conclusion

Disease-associated objects (strains, QTL and genes) have been analyzed using the RGD in-house tool RatMine and the DAVID functional annotation tool. Strains and QTL associated with multiple disease portals have been highlighted in this manuscript. The enrichment of cardio/obesity QTL on rat chromosome 2 has led to the identification of human syntenies housing genes and QTL associated with cardiovascular and obesity diseases. We were able to reciprocally demonstrate the association between diseases and GO annotations in three aspects, BP, CC and MF. The examples presented are meant to validate concepts using curated data. Through these analyses, we hope to encourage the use of public databases and inspire more study by researchers in specific disease areas.

## Supplementary Data

Supplementary data are available at *Database* Online.

Supplementary Data
