# Supplementary material for: Analysis of disease-associated objects at the Rat Genome Database
Source: Database (Oxford). 2013 Jun 21;2013:bat046. doi: 10.1093/database/bat046 (PMC3689439; doi:10.1093/database/bat046)
Supplement: Supplementary Data [file supp_2013_bat046_index.html]

Analysis of disease-associated objects at the Rat Genome Database — Supplementary Data 

# Analysis of disease-associated objects at the Rat Genome Database

## 

files

**Files in this Data Supplement:**

- Supplementary Data - xlsx file
